# Dual blockage of both PD-L1 and CD47 enhances the therapeutic effect of oxaliplatin and FOLFOX in CT-26 mice tumor model

**DOI:** 10.1038/s41598-023-29363-9

**Published:** 2023-02-11

**Authors:** Reza Alimohammadi, Ghanbar Mahmoodi Chalbatani, Masoumeh Alimohammadi, Haniyeh Ghaffari-Nazari, Arezou Rahimi, Esmail Mortaz, Nariman Mossafa, Louis Boon, Seyed Amir Jalali

**Affiliations:** 1 Department of Immunology, School of Medicine, Shahid Beheshti University of Medical Sciences, Tehran, 198571-7443 Iran; 2 Tumor Immunotherapy and Microenvironment (TIME) Group, Department of Oncology, Luxembourg Institute of Health (LIH), Luxembourg City, Luxembourg; 3 Department of Immunology, School of Medicine, Tehran University of Medical Sciences, Tehran, Iran; 4 Department of Immunology, Faculty of Medical Sciences, Mashhad University of Medical Science, Mashhad, Iran; 5 Department of Immunology, Faculty of Medical Sciences, Tarbiat Modares University, Tehran, Iran; 6 JJP Biologics, Warsaw, Poland

**Keywords:** Cancer, Cancer microenvironment, Immune cell death

## Abstract

Colorectal cancer is a poorly immunogenic. Such property can be reverted by using ICD. However, ICD inducers can also induce the expression of inhibitory checkpoint receptors CD47 and PD-L1 on tumor cells, making CRC tumors resistant to mainly CD8 T cell killing and macrophage-mediated phagocytosis. In this study, we examined the therapeutic effect of Oxaliplatin and FOLFOX regimen in combination with blocking antibodies against CD47 and PD-L1. FOLFOX and Oxaliplatin treatment lead to an increase in CD47 and PD-L1 expression on CT-26 cells invitro and invivo. Combining blocking antibodies against CD47 and PD-L1 with FOLFOX leads to a significant increase in survival and a decrease in tumor size. This triple combining regimen also leads to a significant decrease in Treg and MDSC and a significant increase in CD8 + INF-γ + lymphocytes and M1/M2 macrophage ratio in the tumor microenvironment. Our study showed triple combining therapy with FOLFOX, CD47 and PD-L1 is an effective treatment regimen in CT-26 mice tumor model and may consider as a potential to translate to the clinic.

## Introduction

Despite years of advanced research, colorectal cancer (CRC) remains one of the most common and aggressive types of solid tumors. As the first line of treatment in advanced CRC, FOLFOX (5-Fluorouracil plus Oxaliplatin) is used routinely as a combined chemotherapy regimen. However, the acquired drug resistance limits their anti-tumor effect; thus, chemotherapy regimens are not given for curative intent^1,2^.

Although blocking the Programmed cell death protein-1 (PD-1)/programmed death ligand-1 (PDL-1) Axis led to a paradigm shift in cancer immunotherapy and showed great potential in cancer treatment, it cannot be generalized to all types of cancers^3,4^. Recent studies emphasize the importance of the tumor microenvironment's immune contexture on heterogeneous response to immune checkpoint blockade (ICB)^5^. CRC, especially microsatellite stable (MSS) tumors, is one of those with a poor response to checkpoint therapy because of its low immunogenicity and low tumor infiltrated CD8^+^ T cells^4,6,7^. CRC patients with microsatellite unstable (MSI) showed higher pre-existing tumor infiltrated CD8^+^ T cells and better response to anti PD-1 therapy^7,8^.

One way to enhance the poor immunogenicity of tumors and CD8^+^ T cells infiltration is to utilize immunologic cell death (ICD) inducer as a chemotherapeutic agent^1,9,10^. FOLFOX regimen and oxaliplatin were both shown to induce ICD effectively, although they increase checkpoint receptors expression in both tumor cells and lymphocytes^1,11^. Among checkpoint receptors, PD-L1 and CD47 are both overexpressed after treatment with FOLFOX and oxaliplatin in patients^1^.

CD47 upregulation, a mechanism that which cancer cell increases their "selfness" plays an important role in inhibiting tumor cells' phagocytosis by macrophages and dendritic cells (DCs). Furthermore, an increase in its expression leads to the blocking of cross-presentation by DCs^12,13^. Higher expression of CD47 is observed in broad types of cancers associated with lower prognosis and an increase in mortality. Blocking of CD47/ signal regulatory protein α (SIRPα) is a new emerging era in cancer immunotherapy^14^. Several clinical trial studies investigate the clinical benefit of this innate immune checkpoint receptor, with some phase I trial published^15,16^. However, it is expected that monotherapies blocking CD47/SIPR-α fail to act as curative treatment, and combining therapy of innate with adaptive ICBs and chemotherapies are in focus^14,17–19^.

In the current study, we sought to investigate the potential of combining therapy using Oxaliplatin (OXP) as an effective ICD inducer and by releasing damage-associated molecular patterns (DAMPs) that act as an intrinsic vaccine with anti-CD47 and anti-PD-L1. We hypothesize that by providing an "eat me" signal and blocking the "don't eat me" signal, through ICD inducer and blocking CD47 respectively, DCs and macrophages would phagocyte tumor cells and induces an immune response. In the effector arm by CD8^+^ T cells, blocking the PD-L1 enhances their effector function and destroys tumor cells. Using CT-26 mouse model, we further examine replacing oxaliplatin with FOLFOX in our designed combining therapy to assess the source of potential synergism. Our data revealed that in CT-26 tumor-bearing mice, FOLFOX in a combination of anti-CD47 and anti-PD-L1 resulted in a significant increase in survival and reduction in tumor size.

## Results

### CD47 and PD-L1 expression change following OXP and FOLFOX treatment

**Figure 1:**
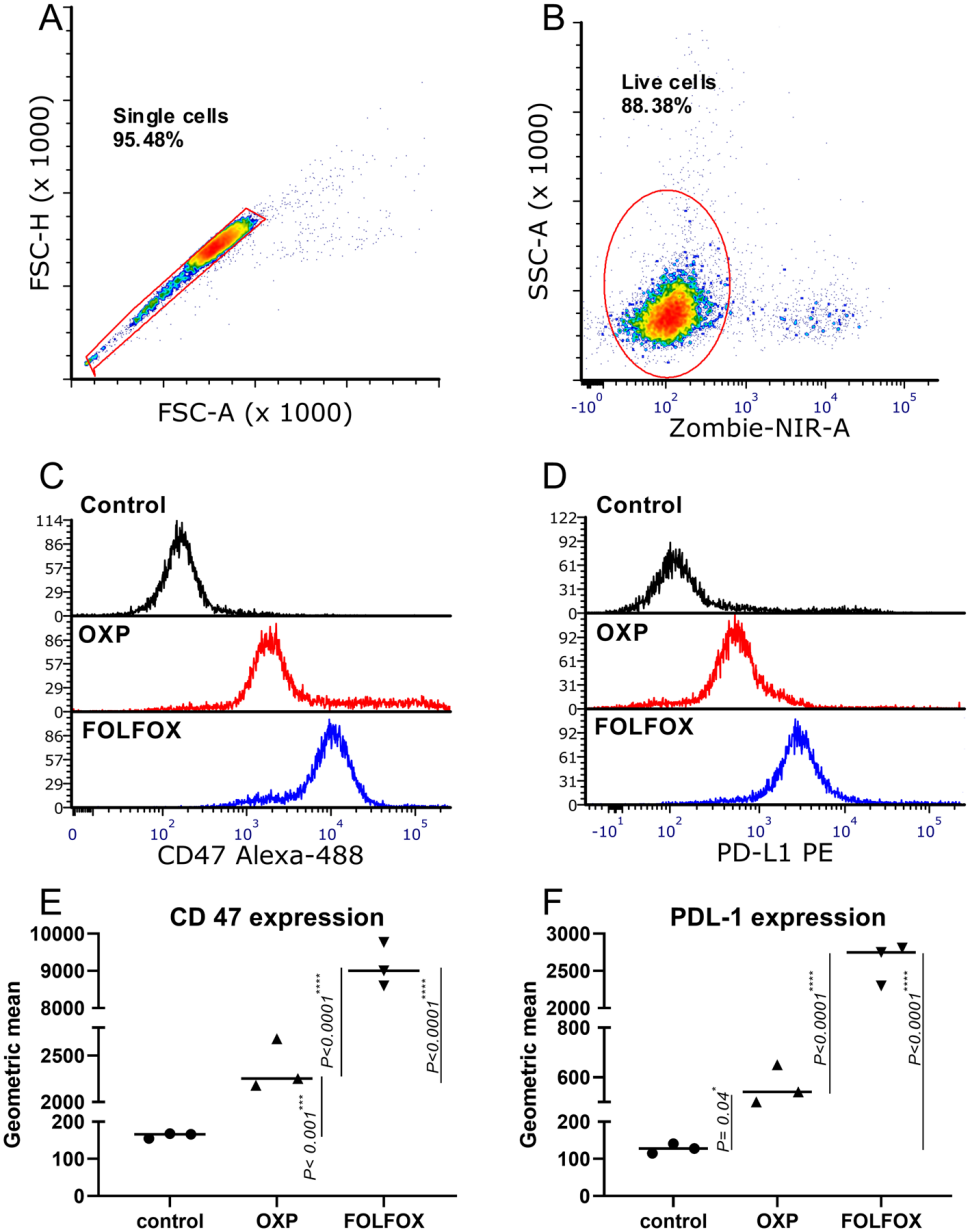
FOLFOX and OXP increased CD 47 and PD-L1 expression on the cell surface of CT-26 cell lines. (**A**–**D**) Gating strategies for analysis of CD47 and PD-L1 cell surface expression in CT-26 tumor cell line following treatment with OXP and FOLFOX. (**E**) CT-26 tumor cells were treated with OXP or FOLFOX, and CD47 expression was analyzed by flowcytometry. (**F**) tumor cells were treated with OXP or FOLFOX, and PD-L1 expression was analyzed by flowcytometry.

To assess whether OXP and FOLFOX regimen affect the expression of CD47 and PD-L1 in CT-26 cells in-vitro, we analyzed their expression by the flow cytometer following treatment with chemotherapeutic agents. Six hours after treatment of cells with either OXP or FOLFOX in the cell culture medium, cells were extracted from wells and analyzed by a flow cytometer. Both OXP and FOLFOX regimen increases the expression of CD47 and PD-L1 on the CT-26 cell surface (Fig. 1A–F). FOLFOX even increases the expression of CD47 and PD-L1 more than OXP (*P* < 0.0001).

**Figure 2:**
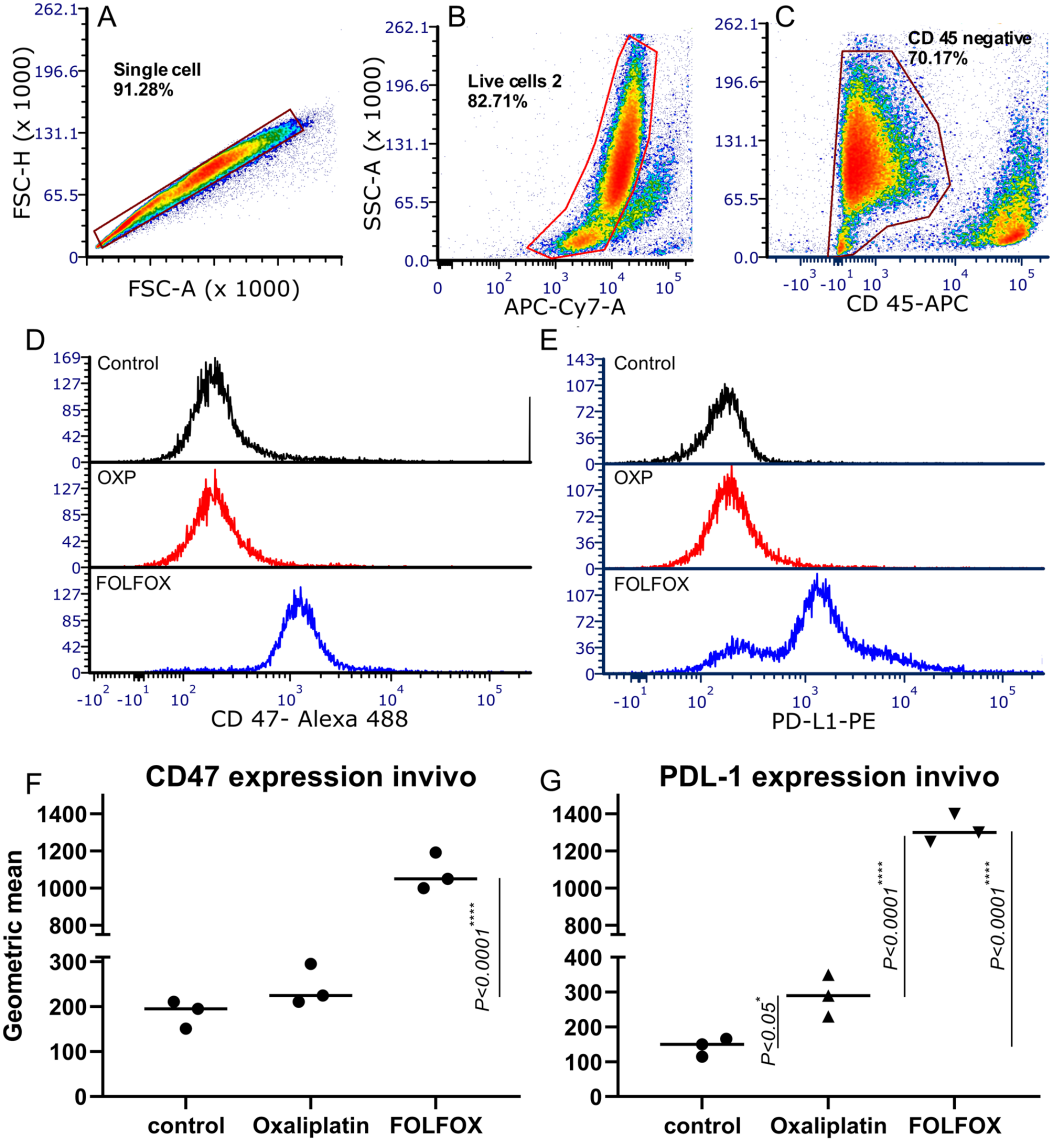
Effect of FOLFOX and OXP on cell surface expression of CD47 and PD-L1 in CT-26 inoculated tumors. (**A**–**E**) Gating strategies for analysis of CD47 and PD-L1 cell surface expression in CT-26 tumor in mice following treatment with OXP and FOLFOX. (**F**) CT-26 tumor cells were treated with OXP or FOLFOX, and CD47 expression was analyzed by flowcytometry. (**G**) Tumor cells were treated with OXP or FOLFOX, and PD-L1 expression was analyzed by flowcytometry.

We next examined the effect of the OXP and FOLFOX regimen on CD47 and PD-L1 expression Invivo (Fig. 2A–G). Mice bearing CT-26 tumor, treated either with OXP or FOLFOX, and 3 days after treatment, mice were euthanized, and single-cell were obtained from tumor bulk. FOLFOX regimen and OXP significantly increased PD-L1 expression in tumor cells extracted from mice (*P* < 0.0001 and* P* = 0.048, respectively). furthermore As shown in Fig. 2C, FOLFOX also increases PD-L1 expression compared to OXP. CD47 expression only significantly increased when mice were treated with FOLFOX (*P* < 0.0001), but OXP failed to increase CD47 expression significantly.

### Survival and tumor size

**Figure 3:**
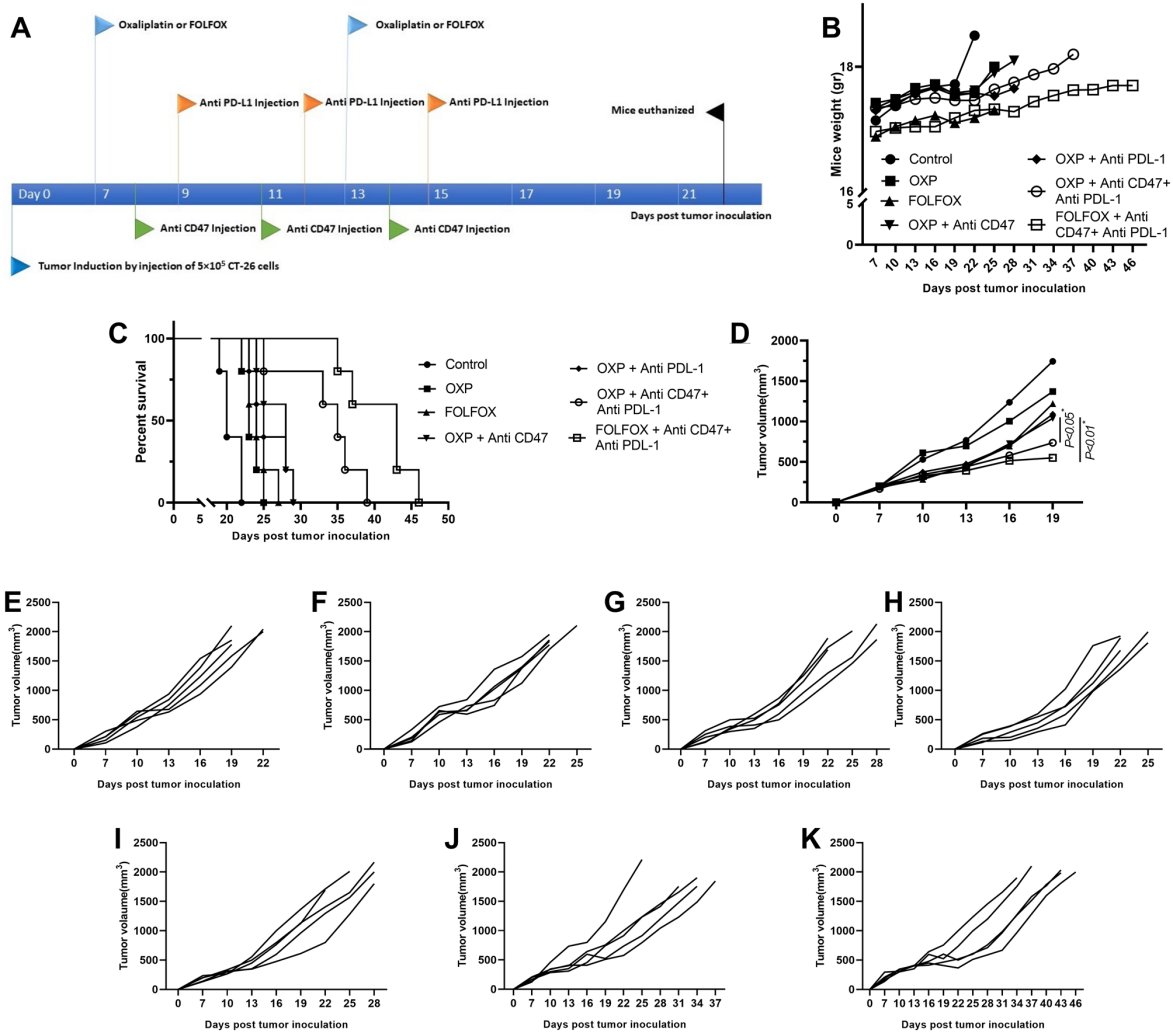
The combination of FOLFOX, anti-CD47, and anti-PD-L1 showed the best potential in the syngenic CT-26 tumor model. (**A**) Detailed schedule for the injection of therapeutic agents. (**B**) Changes in the average body weight (g) of tumor-bearing mice in control and different treated groups. (**C**) Survival of the mice in days, with median survival shown. Significances were determined by log-rank (MantelCox) test; n = 5 mice per group. (**D**) BALB/c mice bearing with CT-26 tumor were injected with corresponding therapeutic regimen according to the indicated schedule. Tumor volume was measured at the indicated time points. (**E**–**K**) tumor volume of each mice in all therapeutic groups.

Next, we aimed to determine whether combining therapy with anti-CD47, anti-PD-L1, and immunological death inducer chemotherapeutic agents (OXP or FOLFOX) could generate anti-tumor response compared to chemotherapy or bicombinig therapy with chemotherapy and checkpoint blockers. When CT-26 mice tumors model get established, each group received corresponding therapeutic agents. detailed schedule for injection of chemotherapeutic agents and/or anti-CD47and anti-PD-L1 is summarized in Fig. 3A. To monitor side effects and possible toxicity of our combining regimen, mice were weighted during the study. No significant weight loss was seen in any of the therapeutic groups (Fig. 3B).

Combining anti-CD47 and anti-PD-L1 with either OXP or FOLFOX regimen led to a significant increase in survival of mice receiving three combining regimens compared to both monotherapies with chemotherapeutic agents and bicombinig regimen (anti-CD47 or anti-PD-L1 with OXP) (Fig. 3C). This data also supported by time to reach end point (TTE) and % tumor growth delay (TTG) data in Table 1. Tumor size analysis also showed that mice receiving anti-CD47, anti-PD-L1, and FOLFOX had the slowest tumor growth rate (Fig. 3D–K).

**Table 1:** Therapeutic efficacy data of therapeutic and control group mice bearing CT-26 tumor.

Groups (n = 5 for each group)	TTE^a^ (days) ± SD	TGD^b^ (%)	ILS^c^ (%)
Control	21.54 ± 3.46	–	–
OXP	22.25 ± 0.63	3.3	13.5
FOLFOX	25 ± 2.28	16.06	18.4
OXP + anti-CD47	27.49 ± 3.81	27.61	30
OXP + anti-PDL-1	26.81 ± 3.7	24.44	25.2
OXP + anti-CD47 + anti-PDL-1	35.62 ± 8.43	65.34	63.1
FOLFOX + anti-CD47 + anti-PDL-1	44.83 ± 8.21	108.06	98

^a^Time to reach end point.

^b^Tumor growth delay.

^c^Increase Life Span.

### Immune cell analysis in spleen and DLN

**Figure 4:**
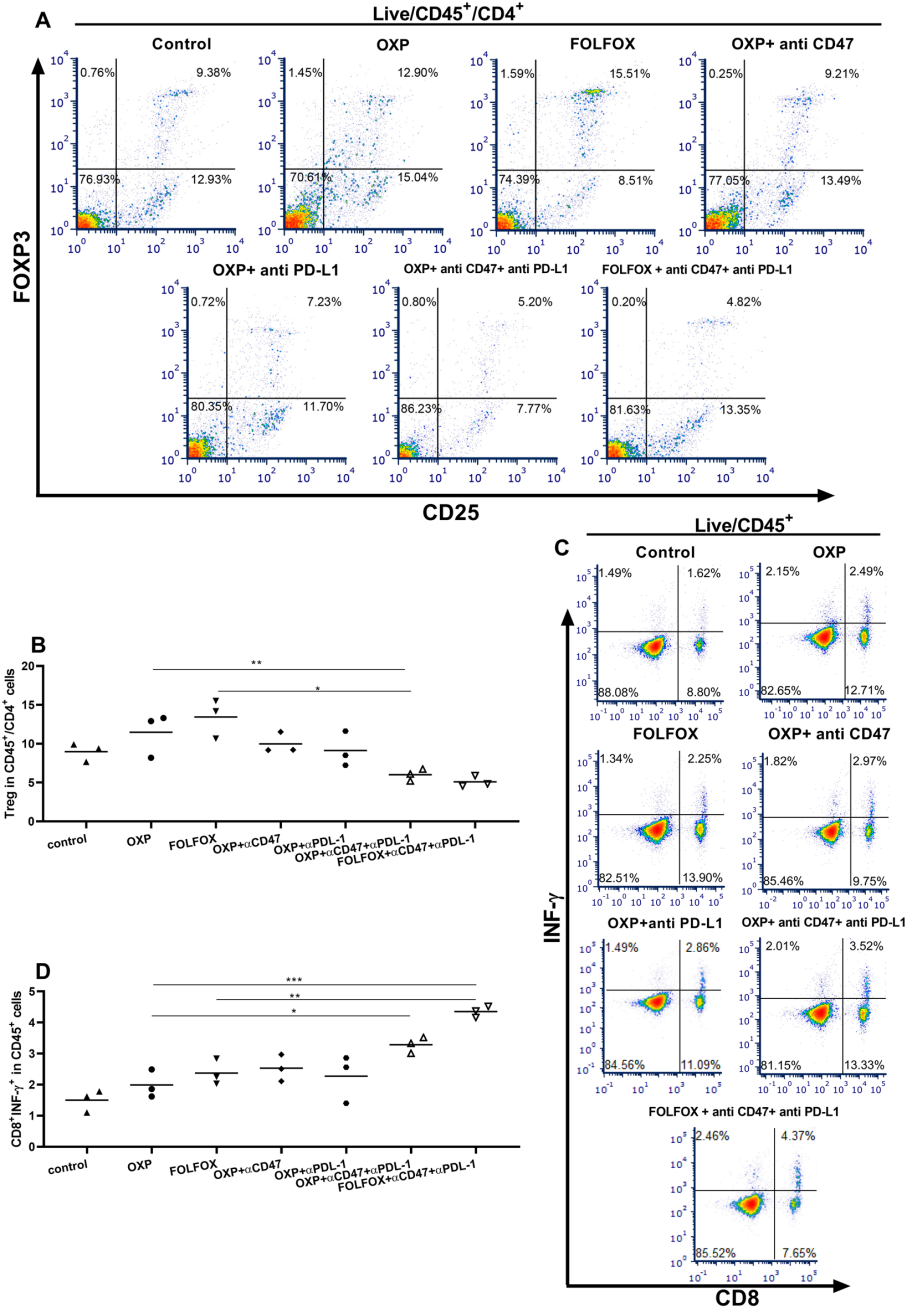
Combining therapy with OXP or FOLFOX with anti-CD47 and anti-PD-L1 deacreased the Treg in CD4^+^ cells and increased CD8^+^/INF-γ^+^cells in CD45^+^ population in spleen. (**A**) Gating strategy used for Treg analysis in spleen. (**B**) OXP and FOLFOX increased Treg in CD4^+^ cells, however this increase wasn’t significant. Both OXP or FOLFOX combined with anti-CD47 and anti-PD-L1 decreased the Treg in CD4^+^ cells, and this decrease was significant compared with mice receiving OXP or FOLFOX regimen alone. (**C**) Gating strategy used for CD8^+^/INF-γ^+^ analysis in CD 45^+^ population of spleen. (**D**) Both OXP or FOLFOX combined with anti-CD47 and anti-PD-L1 increased CD8^+^/INF-γ^+^ in CD45^+^ population. This increase was significant in compared with most of other therapeutic groups.

To determine possible factors that led to better therapeutic response to triple combining regimen in mice bearing CT-26 tumor, we examined the Treg and CD8^+^/INF-γ^+^ T cells content in spleen and lymph nodes. Immune cell profiling in tumor-draining lymph nodes showed no significant change between therapeutic groups (Supplementary Fig. S1). In the spleen, OXP and FOLFOX monotherapy led to an increase in the Treg population, although this increase in the Treg population was not significant. Combining OXP with neither anti-CD47 nor anti-PD-L1 doesn't have a significant impact on the Treg population. Both the Triple combining regimen significantly decreased Treg content compared to OXP and FOLFOX monotherapy (Fig. 4A,B). CD8^+^/INF-γ^+^ T cells also increase in the spleen of mice receiving OXP or FOLFOX + anti-CD47 and anti-PD-L1 compared to mice in the control group and mice receiving OXP or FOLFOX. Furthermore FOLFOX + anti-CD47 and anti-PD-L1 increased CD8^+^/INF-γ^+^ T cells compared to OXP + anti-PD-L1 (Fig. 4C,D).

### Immune cell analysis in the tumor microenvironment

**Figure 5:**
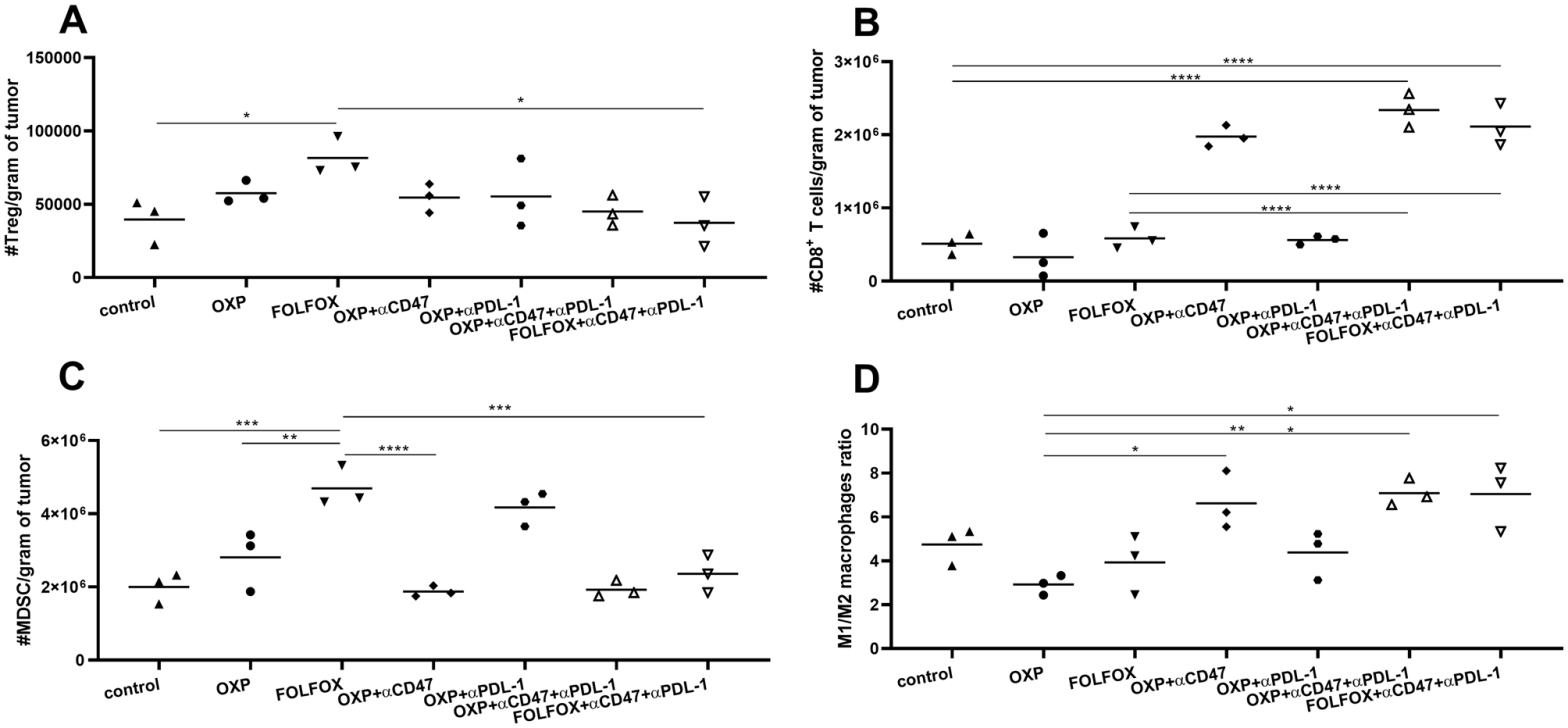
Immune cell analysis in the tumor microenvironment of mice bearing CT-26 tumor. (**A**) Treg number per gram of tumor. Treg number increased in tumor microenvironment following treatment with both OXP and FOLFOX regimen, however, it was only significant in FOLFOX receiving group. Combining anti-CD47 and anti-PD-L1 with FOFOX leads to a significant decrease in Treg per gram of tumor compared to mice receiving FOLFOX alone. (**B**) CD8^+^Tcells number per gram of tumor. OXP and FOLFOX and FOLFOX didn’t change, CD8^+^Tcells number, but in groups which received anti-CD47 as part of their treatment CD8^+^ T cells number per gram of tumor increased significantly. (**C**) MDSC number per gram of tumor. (**C**) OXP, and FOLFOX increased MDSC number however it was only significant in FOLFOX receiving group when we added anti-CD47 in the combining regimen, it leads to a significant decrease in MDSC number compared to mice receiving OXP, FOLFOX and OXP + PD-L1. D, M1/M2 macrophage ratio in the tumor microenvironment. Adding anti-CD47 to the combining regimens, increase the M1/M2 macrophage ratio compared to other groups.

Based on the evidence mentioned above, it is highly likely that combination therapy led to a change in the tumor microenvironment in favor of immune system activation. So we investigated change in different immune cell poplations in the tumor microenvironment following treatments. Treg number per gram of tumor only increased following monotherapy with chemotherapies, but this increase in Treg content was significant in mice receiving FOLFOX. When anti-CD47 and anti-PD-L1 administered simultaneously with both OXP and FOLFOX, it leads to a decrease in tumor Treg content compared to monotherapy with chemotherapeutic agents. However, this decrease in Treg content was only significant in mice receiving FOLFOX + anti-CD47 and anti-PD-L1 compared to mice receiving FOLFOX alone (Fig. 5A).

CD8^+^ T cells per gram of tumor increased in anti-CD47 manner; almost every therapeutic group which received anti-CD47 in the therapeutic regimen showed a significant increase in CD8^+^ T cells compared to groups which didn't receive it (Fig. 5B). MDSC per gram of tumor also increased significantly following OXP and FOLFOX treatments. Furthermore, FOLFOX monotherapy increased MDSC content in tumors compared to OXP monotherapy. This increase in MDSC per gram of tumor was reversed by using anti-CD47 in consistent with its effect on Treg content in the tumor (Fig. 5C). We also investigate the effect of a therapeutic regimen in the M1/M2 ratio in the tumor microenvironment. FOLFOX or OXP monotherapy doesn't affect the M1/M2 ratio in a tumor significantly. But in mice receiving anti-CD47 in the form of bicombinig therapy with OXP and tricombinig therapy with OXP or FOLFOX, the M1/M2 ratio increased significantly (Fig. 5D).

## Discussion

In this study, we evaluated the potential of combining chemotherapy with blocking both innate and adaptive immune check point receptors in the CT-26 mouse model. The importance of combining both innate and adaptive checkpoint blockers was emphasized in several published reports. The rational behind combining innate with adaptive checkpoint blockers is to orchestrate DC, Macrophage, and T cell responses against tumor cells^20,21^. Both OXP and FOLFOX regimens are reported as effective ICD inducers and are in the clinic for CRC patients' routine treatment.

Consistent with numerous studies that reported an increase in checkpoint receptors following chemotherapy treatment, we find an increase in the CD47 and PD-L1 expression following treatment with OXP and FOLFOX^11,22^. Due to the toxicity of the OXP and FOLFOX chemotherapy regimen, the in vitro CD47 and PD-L1 expression analysis could only be assessed six hours after the CT-26 cells were treated. Considering this limitation, PD-L1 expression increased by both OXP and FOLFOX in both in vitro and in vivo experiments, and an increase in the CT-26 cell surface expression of CD47 was found following treatment with OXP and FOLFOX, in vitro. In tumor-bearing mice, only FOLFOX induced an increase in the CD47 expression, and even while OXP treatment increased CD47 expression on CT-26 cells surface, this increase was insignificant. Several studies have revealed that enzymatic digestion has an influence on the expression of cell surface markers; therefore, the absence of a significant increase in the expression of CD47 by OXP may be attributed to a partial loss of CD47 after the enzymatic digestion of the tumor^23,24^. Several mechanisms could be involved in the increase of checkpoint receptor expression after chemotherapies. In a recent study, Samanta et al. reported a HIF-dependent mechanism for an increase in PD-L1 and CD47 expression after treatment with chemotherapeutic agents^11^. Furthermore, ICD inducer chemotherapies increase the infiltration of T cells and the INF-γ secreted by infiltrated lymphocytes. This increase in the infiltration of effector cells and INF-γ secretion suggested to be another factor that increases the inhibitory checkpoint expression, such as PD-L1 and CD47on tumor cells^25–28^.

Both anti-CD47 and anti-PDL-1 were used in combination with different chemotherapy regimens and showed promising results in mice and clinical trials. However, our study is the first to investigate the possible synergy using triple chemo and immunotherapy combination. OXP combined with anti-CD47, and anti-PD-L1 increased survival of mice and reduced tumor growth compared to OXP alone. This result is consistent with an increase in the expression of CD47 and PD-L1 after OXP treatment leading to a suppression of the activation phase and effector phase of immune response. By using anti-CD47 and anti-PD-L1, we were been able to suppress the negative checkpoint signal and orchestrated an immune response against tumor. FOLFOX even showed more potential when used in combination with anti-CD47 and anti-PD-L1. The reason behind this observation could be stronger DAMP release by tumor cell after chemotherapy with FOLFOX over OXP, which was reported by Dosset et al.^1^.

As we expected, our treatment protocols significantly impacted the immune cell profile in the spleen and tumor microenvironment. In the spleen, both OXP and FOLFOX regimen increased the Treg frequency; however, this increase wasn't statistically significant. But when we used anti-CD47 and anti-PD-L1 combined with OXP or FOLFOX resulted in a significant decrease in Treg frequency compared to treatment with OXP and FOLFOX alone. We did not observe this decrease in Treg when mice were treated with OXP in double agent combined with anti-CD47 or anti-PD-L1. In the tumor microenvironment, the change in Treg number per gram of tumor followed the same pattern as the change in Treg frequency in the spleen. But only FOLFOX made statically significant changes. Both anti PD-L1 and anti CD47 have been able to suppress Treg function and reduce Treg number in tumor and lymphatic organs in previous studies^29,30^. In our study, their beneficial effect was only observed when the anti-CD47 and anti-PD-L1 were used in a triple combination regimen with chemotherapeutic agents.

We showed that CD8^+^ T cells infiltration significantly increased in the tumor microenvironment of mice receiving anti-CD47 as one of their treatment regimens. In recent studies, CD47/SIPR-α blockade led to an increase in CD8^+^ Tcells frequency and function in the tumor microenvironment^30,31^. Liu et al. reported that in CD47 blockade, the anti-tumoral effect depends on the cross-priming of CD8^+^ Tcells by DC cells^32^. An increase in CD8^+^ T cells number per gram of tumor in the current study supports the survival benefit and the decrease in tumor size results, except we did not observed the benefit in the survival of mice receiving OXP and anti-CD47. OXP and anti-CD47 failed to reduce Treg content in the spleen and tumor microenvironment; this failure could be the reason why in this treatment strategy, despite an increase in CD8^+^ T cells, we didn't see the therapeutic response.

Besides their cytotoxic effect, chemotherapeutic agents increase MDCS frequency through the action of inflammatory mediators, including GM-CSF, G-CSF, IL1β, IL6, and CCL2^33,34^. We also analyze the MDSCs number per gram of tumor and M1/M2 ratio in the tumor microenvironment. MDSC number per gram of tumor increased following monotherapy with both OXP and FOLFOX regimen. When we add anti-CD47 in the combining therapies regimen, this increase in MDSCs reversed, and the MDSC number per gram of tumor decreased. Consistent with our study Wu et al. also reported a decrease in the MDSC population following treatment with anti-CD47^35^. M2 macrophages play an important role in cancer progression by providing an immunosuppressive microenvironment for tumor growth^36^. Zhang et al. reported M2 macrophages' repolarization to M1 following anti-CD47 treatment^37^. In our study in three mice groups receiving anti-CD47 in combining regimen treatments, the M1/M2 macrophages ratio were increased.

Our results validated the concept of using both innate and adaptive checkpoint blockers in combination with chemotherapeutic agents. Based on numerous studies, among different chemotherapeutic agents, those with ICD inducer ability seem to fit best in combination therapies with checkpoint inhibitors. If not for all cancers, at least in CT-26 tumor model it seems that blocking both CD47 and PD-L1 axis needed for the best therapeutic response.

## Methods

### Antibodies and reagents

The IgG anti-mouse CD47 and IgG anti-mouse PD-L1 blockade monoclonal antibodies (Clone: MIAP301 and 10F.9G2 respectively) were purchased from Bioxcell (NH, USA). PerCP Rat anti-mouse CD4 antibody (RM4-5 clone), PE-labeled Rat anti-mouse CD8a antibody(53-6.7 clone), PE-labeled Rat anti-mouse CD25 antibody (PC61 clone), Alexa-flour^®^ 488 labeled Rat anti-mouse Foxp3 antibody (MF-14 clone), Alexa Fluor^®^ 488 anti-mouse CD47(MIAP301), PE anti-mouse CD274 (B7-H1, PD-L1) antibody (10F.9G2 clone), PerCPanti-mouse/human CD11b antibody (M1/70 clone), Alexa Fluor^®^ 488 anti-mouse CD80 antibody (16-10-A1 clone), PE anti-mouse CD206 (MMR) antibody (C068C2 clone), APC labeled anti-mouse CD45 antibody (30F11 clone), NIR Zombie die (for live/Dead discrimination), as well as appropriate isotype control antibodies and a True-Nuclear™ transcription factor buffer set were purchased from Biolegend (CA, San Diego). Collagenase type I was purchased from Gibco (NY, USA). DNase type I was purchased from Roche (USA). The remaining chemical solvents and reagents were chemical grade.

### Invitro procedures

To determine the effect of OXP and FOLFOX on cell surface expression of CD47 and PD-L1, CT-26 cell line (Pasture Institute, Tehran, Iran ) were grown in RPMI 1560 Gibco (NY, USA) with 10% FBS Gibco (NY, USA), penicillin and streptomycin then seeded to 6-well plate. After 24 h cell were treated either with 50 µM OXP (Sobhan, Tehran, Iran) or 50 µM OXP (Sobhan, Tehran, Iran) plus 10 µM 5-fluorouracil (FOLFOX regimen), (Alhavi, Tehran, Iran). The concentration of chemotherapeutic agent used for the in-vitro study was based on previous studies, which led to maximum ICD induction^1^. Six hours after CT-26 tumor cells were treated with chemotherapeutic agents, cells were harvested, stained with fluorochrome-conjugated antibodies, and analyzed for cell surface expression of CD47 and PD-L1 with BD FACSlyric flow cytometer (Becton Dickinson, USA).

### Mice and in vivo procedures

Six to eight weeks old female BALB/c mice were purchased from Pasteur Institute, Thran, Iran and housed in proper condition. All animal studies were carried out in accordance with relevant guidelines and regulations and ARRIVE guidelines. The animal study also approved by the Institutional Ethical Committee and Research Advisory Committee of Shahid Beheshti University of Medical Sciences with ethic code number: IR. SBMU. MSP.REC.1396.370. A detailed tumor induction procedure is described previously^38,39^. Briefly, 10^6^ CT-26 tumor cells were injected into the right flank of each BALB/c mice. When the tumor reached 150 mm^3^, mice were randomly assigned into treatment groups based upon their tumor's volume, as follows: Control (mice receiving placebo), OXP (mice receiving Oxaliplatin, 6 mg/kg), FOLFOX (mice receiving Oxaliplatin, 6 mg/kg + 5FU, 50 mg/kg + Flavinin 90 mg/kg), OXP + anti-CD47 (100 µg per mice), OXP + anti-PD-L1 (200 µg per mice), OXP + anti-CD47 + anti-PD-L1 and FOLFOX + anti-CD47 + anti-PD-L1. The dose and schedule used for OXP and FOLFOX regimen were based on previous studies, with maximum ICD induction and least cytotoxicity^1^. We started chemotherapy agents injections after tumors reached 150 mm^3^ (7 days after tumor inoculation). The detailed schedule for the injection of therapeutic agents is illustrated in Fig. 3A. The tumor size of mice bearing CT-26 tumor was measured with digital calipers every three days, and tumor volume was calculated as a × b^2^/2, where a is the largest diameter and b the smallest diameter. Tumor size measurements were continued until tumor size reached 2000 mm^3^. The detailed calculation for time to reach the endpoint (TTE), tumor growth delay (%TGD), and increased life span (ILS) of each mouse was described in our previous studies^40,41^.

### Flow cytometry analysis

Twenty-two days after tumor inoculation (7 days after the last injection in therapeutic groups), three mice from each group were sacrificed by CO_2_ asphyxiation under Isoflurane euthanasia, and their draining lymph node, spleen, and tumors were analyzed for the immune cell population. In the case of draining lymph nodes and spleen, the frequency of Treg and CD8^+^/INF-γ^+^ T cells were analyzed in the CD45^+^ live cell population. For single-cell separation, we used a 70 µm cell strainer (SPL, South Korea), and all staining procedures for flow cytometry were based on manufacturer instructions. For tumor analysis, we digested the tumor with 2 mg/ml collagenase type I and 10 IU/ml DNase type I in the RPMI medium. After 90 min of digestion, the single cells were separated from cell suspension using a 70 µm cell strainer. To analyze the effect of Oxaliplatin and FOLFOX on cell surface expression of CD 47 and PD-L1 Invivo, their expression was analyzed in CD45^-^ live cell gate. We also analyzed the exact population of T reg, CD8^+^/INF-γ^+^ T cells, MDCS, and M1/M2 macrophages in the tumor microenvironment. To quantify the exact number of infiltrated cells, we calculated the separated cells per gram of tumor. Using the frequency of each cell population in the live cell's gate, we were able to change the frequency of each cell population to the number of specific cells per gram of tumor. The gating strategy used for each cell population is summarized in the corresponding figures.

### Statistical analysis

Statistical analysis was performed using GraphPad Prism software (CA, USA). For two-group comparisons, the t-test and the Mann–Whitney test were used. For multiple group comparison, the one-way or two-way ANOVA test was used. All differences were considered statistically significant at the level of p < 0.05 (*p < 0.05, **p < 0.01). Analysis of survival was done with a Mantel–Cox test. Values of *p* < 0.05 were considered statistically significant.

## Supplementary Information

Supplementary Figure S1.

## Data Availability

The data that support the findings of this study are available upon request from the corresponding author, Seyed Amir Jalali by email (jalalia@sbmu.ac.ir).
