# Supplementary figures and images for: Dual blockage of both PD-L1 and CD47 enhances the therapeutic effect of oxaliplatin and FOLFOX in CT-26 mice tumor model
Source: Sci Rep. 2023 Feb 11;13:2472. doi: 10.1038/s41598-023-29363-9 (PMC9922272; doi:10.1038/s41598-023-29363-9)

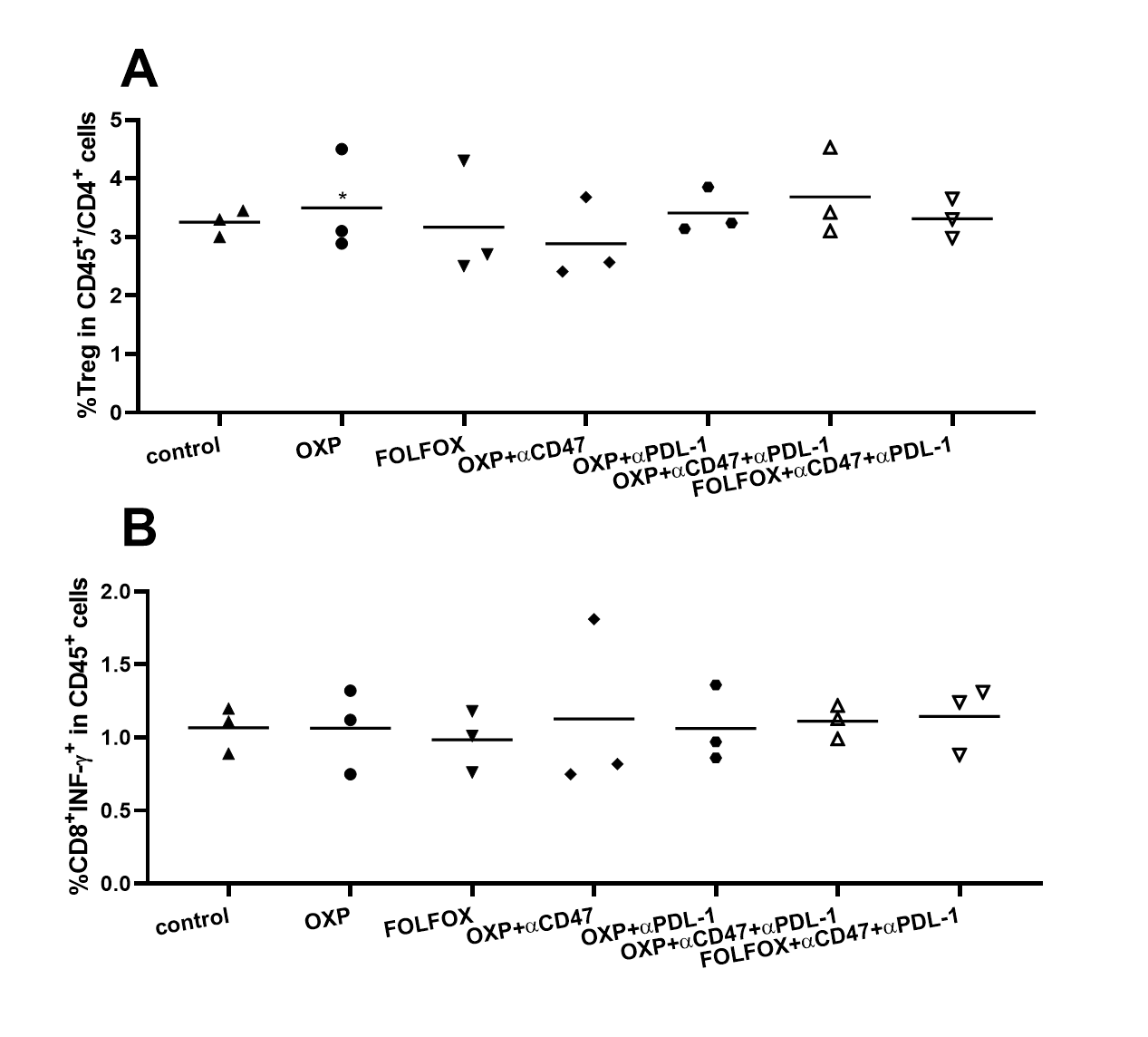


Fig S1. A, the frequency of Tregs in lymph node. B, the frequency of CD8+ T cells in lymph node.

Supplement: Supplementary file 1 — Supplementary Figure S1. [file 41598_2023_29363_MOESM1_ESM.docx]
